# Dual-Band Dual-Beam Shared-Aperture Reflector Antenna Design with FSS Subreflector

**DOI:** 10.3390/s25092934

**Published:** 2025-05-06

**Authors:** Qunbiao Wang, Peng Li, Guodong Tan, Yiqun Zhang, Yuanxin Yan, Wanye Xu, Paolo Rocca

**Affiliations:** 1 State Key Laboratory of Electromechanical Integrated Manufacturing of High-Performance Electronic Equipments, Xidian University, Xi’an 710071, China; 2 National Key Laboratory of Electromechanical Coupling in Electronic Equipment, Xidian University, Xi’an 710071, China; 3 School of Cyber Science and Technology, University of Science and Technology of China, Hefei 230027, China; 4 ELEDIA Research Unit, CNIT—University of Trento, Via Sommarive 9, 38123 Trento, Italy; 5 ELEDIA Research Center (ELEDIA@UniTN—University of Trento), DICAM—Department of Civil, Environmental, and Mechanical Engineering, Via Mesiano 77, 38123 Trento, Italy; 6 ELEDIA Research Center (ELEDIA@XIDIAN—Xidian University), No. 2 South Taibai Road, Xi’an 710071, China

**Keywords:** dual-band dual-beam reflector antenna, FSS subreflector, shaped reflector antenna, shared antenna aperture

## Abstract

In this study, a dual-band dual-beam shared-aperture reflector antenna based on a Cassegrain configuration is designed using a frequency-selective surface (FSS) subreflector. The antenna generates two shaped beams that operate at different frequencies and can spatially overlap. One beam contour can be independently optimized by properly designing the shape of the main reflector. The contour of the second beam is defined by optimizing the unit cell and geometry of the FSS-based subreflector once the shape of the main reflector is set. The reflector antenna design is cast as the optimization of a suitably defined cost function aimed at yielding the desired directivity performance in the regions of coverage. In order to validate the proposed solution, a set of numerical experiments was conducted using most of China and Shaanxi province as benchmark examples.

## 1. Introduction

With the growing interest in and development of space technologies and applications, such as satellite communications, navigation, remote sensing, and deep space exploration [1], more challenging requirements are imposed on antenna performance [2]. In order to increase satellite communication capacity and meet multi-mission requirements [3], multiband [4] and multibeam [5] antenna technologies are of interest and have been widely studied since the 60s. Among the current trends, one of the main demands is for satellite systems to have the capability to simultaneously provide multiple coverages in multiple bands [6] in order to reduce the volume required on the launcher fairing and spacecraft, thereby limiting costs. Meanwhile, it could also mitigate the congestion issue in the space environment [7].

To this end, sharing the reflector antenna aperture to synthesize differently shaped beams is an option [8]. There are two typical approaches to designing a reflector antenna with a shared aperture. One is to design the feed system to expand the antenna capability by introducing beam forming networks (BFN) that can optimize the excitation coefficients [9] or the array feed geometrical arrangement [10] to generate multiple beams with different contours. However, it is obvious that the BFN will be very complex in this case, which further increases the burden and manufacturing costs of the satellite. Moreover, as the frequency increases, the radio-frequency losses increase [11]. Another design method considers the change in the reflector surface shape [12]. Thus, the phase distribution of the electromagnetic waves in the aperture plane is adjusted, thereby transforming the pencil beam obtained with a classical reflector antenna into a contour beam. Compared with the BFN method, this latter approach has the advantages of obtaining a reflector antenna solution with a lower cost, lighter weight, and higher aperture efficiency.

The key to the second approach is the design of the shape of the reflector. The earliest method for generating the shape of the reflector surface was the wavefront synthesis method proposed in 1975 [13] based on geometrical optics (GO). Now, reflector synthesis techniques have formed a relatively mature theoretical system and have been widely used in electromagnetic missions [14]. The emergence of the design of the shape of the reflector not only brings greater flexibility and reliability to communication systems but also opens up new possibilities for the design of satellite communication equipment.

**Figure 1:**
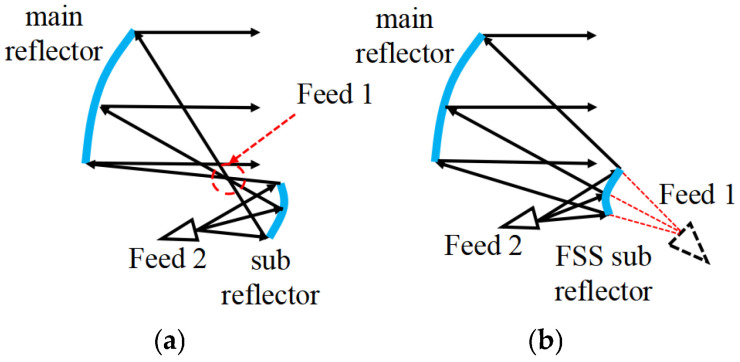
Two reflector antenna configurations: (**a**) Gregorian and (**b**) Cassegrain.

A triple-reflector system was proposed in [15] to generate and control two beams using two subreflectors. Although this solution eliminates the disadvantages associated with the presence of the BFN, it is only suitable for spatially isolated beams and requires more volume because it is equipped with two subreflectors, thus limiting its applicability in satellites. In [16], a shaped reflector with an array feeder was used to generate multiple beams. Although this solution is more compact, its efficiency is lower than that of independent-shaped reflector apertures. A hybrid reflector antenna based on a Gregorian configuration with two feeds, one illuminating the main reflector surface only and the other jointly illuminating the main and subreflector surfaces, was introduced in [17] to produce two different contour beams. The main issue is that this method can only produce spatially isolated beams due to the fact that the first feed must be offset in order to avoid obstructing the beam formed by the second feed, as shown in Figure 1a. Moreover, since the beam generated by the second feed is focused on one point reflected by the subreflector, thermal issues, and arc discharge problems can easily arise.

A frequency-selective surface (FSS) can be exploited to fit the antenna radiation requirements for a specific frequency band by adjusting the transmissions and reflections of a properly designed periodic structure [18]. In recent years, FSS technology [19] has been increasingly used in reflector antennas [20] because it can provide an additional degree of freedom for the development of novel shared-aperture reflector antennas. In this framework, the design of a dual-band dual-beam shared-aperture reflector antenna based on a Cassegrain configuration is addressed in this work, where the use of an FSS-based subreflector is taken into account, as shown in Figure 1b. More specifically, the first beam is generated by a feeder located behind the subreflector and illuminates the main reflector through the FSS-designed surface. The second beam is generated on a different band by a feeder located in front of the subreflector, exploiting the reflection of the FSS to illuminate the main reflector. The shapes of both the main reflector and subreflector are optimized to generate a radiation power pattern covering the service area and provides the desired gain and operational bandwidth to maximize the energy utilization rate [21].

To the best of the authors’ knowledge, the main advantages of the proposed solution over the existing literature are as follows: (1) the design of a more compact structure compared to the use of a dual subreflector system [15]; (2) the achievement of higher aperture efficiency with respect to an array-fed shaped reflector system [16]; (3) compared to the hybrid reflector system [17], since the Cassegrain configuration antenna electromagnetic energy does not converge at one point as Gregorian does, it has no thermal issues and arc discharge problems, and when Feed 1 is offset, it can work like [17], while Feed 1 is not offset, it has the possibility of generating spatially non-isolated beams; and (4) the generation of contour beams with more uniform directivity values compared to [22].

The remainder of this paper is organized as follows. Section 2 describes the antenna concept and the formulation of the dual-band dual-beam shared-aperture reflector antenna design problem. Section 3 focuses on the customization and characterization of the FSS and discusses the results obtained. Section 4 concludes the paper.

## 2. Design Concept and Theory

A. Multiband Multibeam Antenna Concept

**Figure 2:**
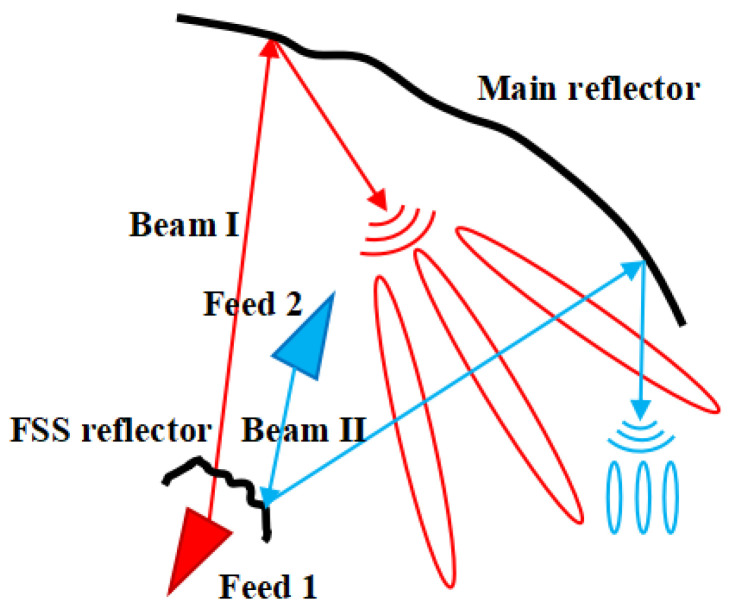
Antenna-shaped beam.

Dual-reflector antennas generally have two focal points, but usually, only one focal point is used to set up the feed. A hybrid reflector antenna [17] was previously designed based on the Gregorian configuration, but it could only produce spatially isolated beams. With the development of FSS technology, it is possible to set up two feeds with different operating frequencies in order to generate different beams. Additionally, Ref. [23] verified that the introduction of an FSS does not change the original antenna radiation power pattern, providing new options for designs. As shown in Figure 2, if Feed 1 and Feed 2 are positioned at two different focal points of the Cassegrain antenna configuration, Beam I can pass through the FSS reflector, and Beam II is reflected by the FSS reflector; therefore, two different beams can be easily obtained. Furthermore, by changing the shape of the two reflector surfaces [24], the beam coverage of the area of concern can be obtained. It is worth mentioning that Feed 1 can be either offset or not offset, and the two reflectors can also use offset surfaces or standard surfaces (not offset). When Feed 1 is offset, the beam generation is similar to that in [17], and spatially isolated beams can be produced. Figure 2 shows the generation of spatially overlapping beams when Feed 1 was not offset. Moreover, due to the research attention that has been paid to the reflector, the shape of the reflector designed by the shaping method can now be machined in detail [11].

It is evident that the contour of Beam I is only related to the shape of the main reflector. However, the contour of Beam II is related to both the main and subreflectors. Therefore, during the shaping process, the main reflector is shaped first to obtain a contour for Beam I that meets the requirement of the effective isotropic radiated power value in the desired coverage service area [25]. Then, the subreflector is shaped while maintaining the shape of the main reflector surface unchanged to obtain a contour for Beam II that meets the directional requirements in another coverage area.

**Figure 3:**
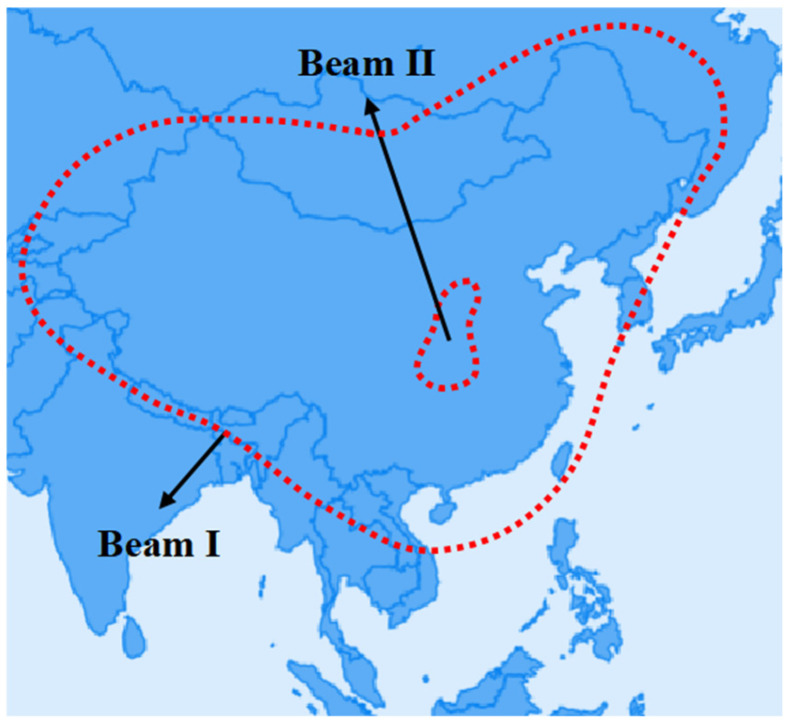
Antenna coverage.

In this paper, Beam I is designed to operate in the S-band (2.0–4.0 GHz), covering most of continental China for communication. Beam II operates within the Ku-band (12.0–16.0 GHz) to cover the Shaanxi area. Figure 3 shows the coverage. Because the Shaanxi area is covered by both beams in this example, it can use two frequency bands to communicate and ensure uninterrupted communication if one band is interfered with.

B. Shaped Theory

**Figure 4:**
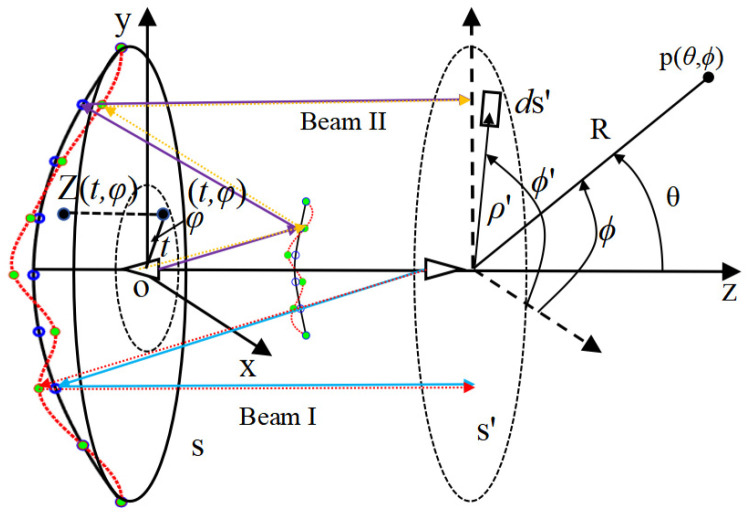
Schematic of a dual-reflector antenna.

The key to the reflector shaping method is to describe the shape of the reflector surface. After more than three decades of development, Jacobi–Fourier polynomials [26] have become the standard method for representing shaped reflectors and serve as a reference method for validating new technologies. In Figure 4, the shape of the reflector can be described by Z(x, y) in the Cartesian coordinate system XYZ, where the origin is the phase center position of one feed of the dual-reflector antenna feeds. The Jacobi–Fourier polynomials method involves first changing this description to a description in terms of the parameters *t* and *φ*, where *t* is the polar diameter divided by the radius of the reflector aperture, and *φ* is the polar angle, and then further describing Z(*t*, *φ*) using the Jacobi–Fourier orthogonal function expression (see Equation (1)).

(1) $$Z\left(t,\varphi \right)=\sum_{n=0}^{N}\sum_{m=0}^{M}\left(c_{nm}\cos n\varphi +d_{nm}\sin n\varphi \right)F_{m}^{n}\left(t\right)$$

(2) $$\begin{array}{l} F_{m}^{n}\left(t\right)=\sqrt{2\left(n+2m+1\right)}P_{m}^{\left(n,0\right)}(1-2t^{2})t^{n}\\ 0\leq t\leq 1,\;0\leq \varphi \leq 2\pi \end{array}$$

where *M* and *N* are constants that determine the number of parameters, control the shape of the reflector surface, and determine the number of *c_nm_* and *d_nm_*. The larger the values of *M* and *N*, the more up-and-down deformations can be obtained on the reflector; however, this also increases the optimization time and computational resources required. In this paper, *M* and *N* are set to 4 and 7, respectively, the same values used in [25]. In Equation (2), $P_{m}^{\left(n,0\right)}(1-2t^{2})$ is a Jacobi polynomial. The entire computational procedure can be found in [26], and the initial value selections for *c_nm_* and *d_nm_* for the parabolic reflectors can also be found in [26].

In addition, Jacobi–Fourier polynomials for hyperbolic surfaces are introduced. As shown in Figure 4, the z-coordinate of a point on a sub-hyperbolic reflector can be expressed in terms of parametric *t* and *φ*, as shown in Equation (3):

(3) $$Zs\left(t,\varphi \right)=\sqrt{a^{2}+\frac{a^{2}r^{2}t^{2}}{b^{2}}}+c$$

where *a* is the length of the real half-axis, *b* is the length of the imaginary half-axis, *c* is the half-focal distance, and r is half the aperture of the subreflectors. The initial values of the Jacobi–Fourier expansion coefficients for the subreflector surface can then be obtained using Equation (4) as follows:

(4) cnmdnm=εn2π∫02π∫01Zst,φcosnφsinnφFmnttdφdtεn=12  n=0n≠0

where $F_{m}^{n}\left(t\right)$ is the same for both the main reflector and the subreflector and is obtained using Equation (2). The details of the calculation procedure can be found in ref. [26]. Finally, by substituting the initial values of *c_nm_* and *d_nm_* into Equation (1), the ideal surfaces of the two reflectors can be obtained, and by optimizing the values of *c_nm_* and *d_nm_*, the shapes of the two reflector surfaces can be changed.

As shown in Figure 4, the ideal reflector surfaces are all plotted using black lines with blue rings, and the shaped reflector is plotted using red lines with green circles. When only the shape of the main reflector surface is changed, the electromagnetic wave, Beam I, changes from the original blue arrow to the red arrow. S’-plane is the aperture field plane, and the difference between the red line and the blue line is the electromagnetic wave path length difference in Beam I after the main reflector surface has been shaped. Similarly, when the shape of the subreflector surface is changed while the shaped main reflector surface is unchanged, the difference between the purple and orange lines is the path length difference in Beam II. Both path length differences can be obtained by performing the following two steps: Firstly, the z-coordinate of a point on both the ideal and shaped reflector surfaces can be calculated by substituting the initial *c_nm_* and *d_nm_* and optimized *c_nm_* and *d_nm_* into Equation (1), respectively. Secondly, the electromagnetic wave propagation path shown in Figure 4 can be used to obtain the path length difference.

C. Optimization Method

The method for generating the shaped reflector surfaces is described in the previous section. As the shape of the reflector antenna surface changes, the path length difference also changes. Equation (5) can be used to calculate the far-field electric field values *E* [27] from the change in length.

(5) $$E(\theta ,\phi )=\iint_{s}f(\rho^{\prime },\phi^{\prime })e^{j\delta }e^{j\Phi (\rho^{\prime },\phi^{\prime })}\rho^{\prime }d\rho^{\prime }d\phi^{\prime }$$

where E(θ,ϕ) represents electric field value at a point in the far field, $f\left(\rho^{\prime },\phi^{\prime }\right)$ are the amplitude distribution functions in the antenna aperture, and $\Phi \left(\rho^{\prime },\phi^{\prime }\right)$ are the phase distribution functions in the antenna aperture, respectively. Figure 4 shows the meanings of the other parameters more clearly. The S-plane is the main reflector antenna aperture, p(θ,ϕ) is a far-field observation point, $\left(\rho^{\prime },\phi^{\prime }\right)$ is a certain point in polar coordinates on the aperture plane, and δ is the phase error of the antenna aperture field caused by the shaped reflector surface. In δ=kτ, k is the free space wave constant and τ is the path length difference. By partitioning the integral area into V triangular cells [28], Equation (5) can be expressed as follows:

(6) $$E(\theta ,\phi )=\sum_{I=1}^{V}E_{I}(\theta ,\phi )=\sum_{I=1}^{V}f_{I}(\rho^{\prime },\phi^{\prime })e^{j\Phi (\rho^{\prime },\phi^{\prime })}e^{j\delta }\Delta s_{I}$$

where $\Delta s_{I}$ is the area of the I-th cell.

Further, the directivity value *D* in dBi can be calculated using Equation (7) as follows:

(7) $$D=10\log_{10}\left(\frac{4\pi {\left|E(\theta ,\phi )\right|}^{2}}{P_{r}}\right)$$

where $P_{r}$ is the total radiated power.

Accordingly, with the given values of *c_nm_* and *d_nm_* controlling the shape of the reflector surface, the directivity at any far-field point can be calculated using Equation (7). In this paper, we aim to ensure that the minimum values of the directivity of the two contour beams in the coverage area of interest meet the requirements. The minimum value of directivity [17] in the coverage area can be estimated using Equation (8):

(8) $$D_{\min}=10\log\frac{41,253}{S_{c}+67.5\times C_{c}/D_{\lambda }}$$

where *S_c_* is the coverage area of the shaped beam in square degrees, *C_c_* is the coverage perimeter of the shaped beam in degrees, and *D_λ_* is the projected diameter of the antenna reflector in wavelengths. Note that Equation (8) is not only an estimation equation for *D_min_* but also a design equation. The antenna aperture *D_λ_* can be designed when the coverage areas *S_c_* and *C_c_* and minimum directivity *D_min_* are known.

In order to ensure that the optimization problem meets the minimum directivity requirement for the coverage area and to match the contour beam as closely as possible to the coverage area, the following cost function is defined:

(9) $$\Gamma =\frac{1}{N_{1}+N_{2}}\left(\begin{array}{c} \sum_{i=1}^{N_{1}}{\left(D_{i}-D_{\min}\right)}^{2}\times \Theta \left(D_{\min}-D_{i}\right)+\\ \sum_{u=1}^{N_{2}}{\left(D_{u}-D_{\min}\right)}^{2}\times \Theta \left(D_{u}-D_{\min}\right) \end{array}\right)$$

where *N*_1_ is the number of discrete observation sites within and at the boundaries of the coverage area, *N*_2_ is the number of discrete observation sites out of the coverage area, *D_i_* and *D_u_* are the directivity values of the *i*-th or *u*-th discrete observation site, respectively, and $\Theta \left(\cdot \right)$ is the Heaviside function equal to 1 when the argument is positive (i.e., *D_min_* > *D_i_* → the directivity values at *i*-th discrete observation site is below the desired value) and 0 otherwise [i.e., *D_min_* ≤ *D_i_* → the coverage area directivity requirement is fulfilled].

In a previous method [11], the authors used the cost function in Equation (10) without the item *D_u_*, which only required the directional values within the covered area to meet the requirements without considering the directional values outside the covered area. While Equation (9) takes into account directional values outside the coverage area to make the contour beams match the map boundaries better, surprisingly, the addition of item *D_u_* achieves a flat-top beam, resulting in a uniform distribution of fields in the desired region and a rapid reduction in fields outside the region. This advantage can be used to construct a high-intensity radiated field [29].

(10) $$\Gamma_{1}=\frac{1}{N_{1}}\left(\sum_{i=1}^{N_{1}}{\left(D_{i}-D_{\min}\right)}^{2}\times \Theta \left(D_{\min}-D_{i}\right)\right)$$

**Figure 5:**
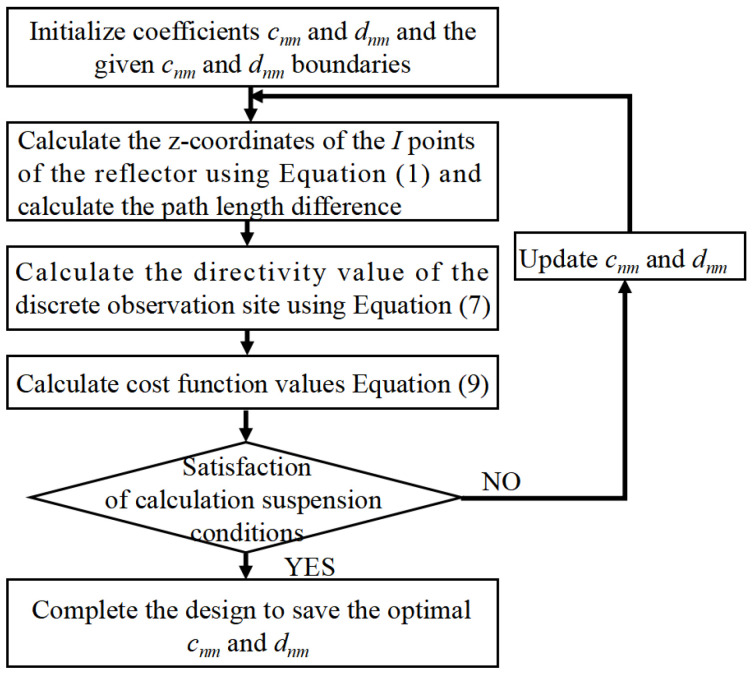
Steps of optimization.

The optimized design can then be easily completed using the steps shown in Figure 5. Here, with the help of particle swarm algorithm (PSO) [30] optimization, multiple sets of *c_nm_* and *d_nm_* coefficients can be performed simultaneously, and directions for updating *c_nm_* and *d_nm_* are provided.

## 3. FSS Selection and Numerical Results

**Figure 6:**
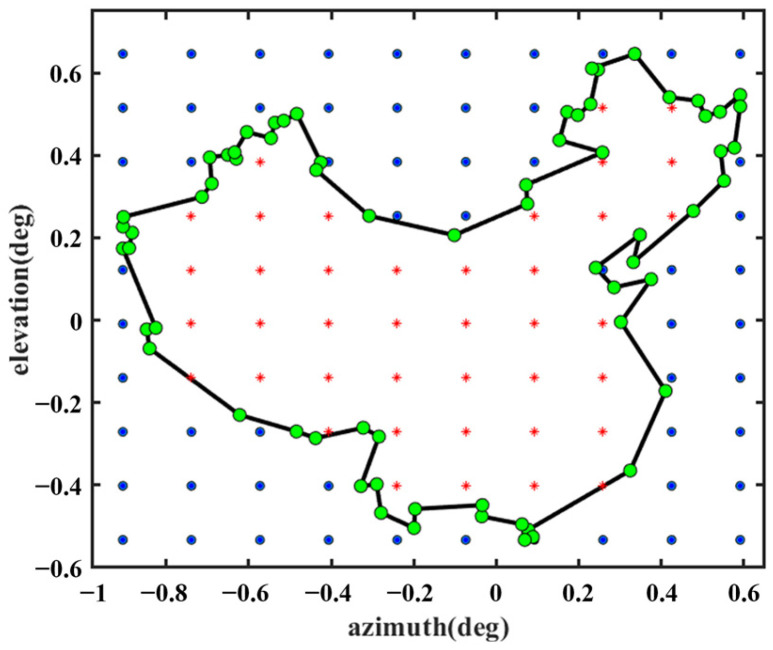
Beam I covers the discrete observation sites.

**Figure 7:**
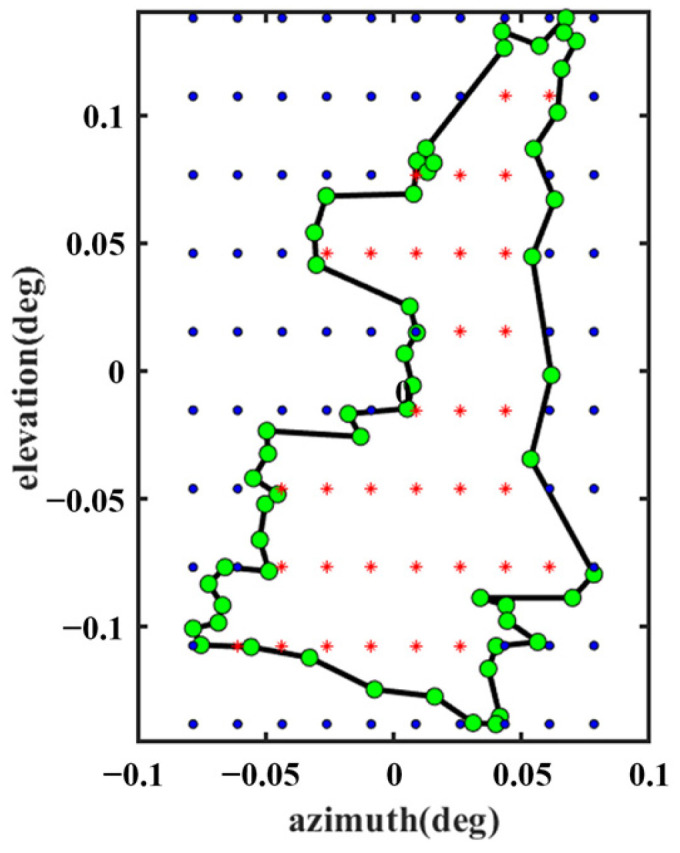
Beam II coverage of discrete observation sites.

For this design, Feed 1 operates in the S-band, and Feed 2 operates in the Ku-band. Thus, the first step is to select the appropriate FSS unit to ensure that the electromagnetic waves from Feed 1 and Feed 2 pass through the subreflector in the correct mode, as shown in Figure 2. The FSS can be redesigned to operate in other bands as needed, and this design is illustrated with S and Ku bands as examples. The second step involves selecting the appropriate parameters of the antenna to design a shaped reflector surface that sufficiently covers the region of interest. For this design, Beam I covers most of continental China, and Beam II covers the area of Shaanxi. The desired observation sampling points, as shown in Figure 6 and Figure 7, were selected as evaluation points for the cost function (see Equation (9)). The green and red points belong to *N*_1_ in Equation (9), and the blue point belongs to *N*_2_ in Equation (9).

In this section, two aspects are discussed: the FSS design and the shaping results.

A. FSS Design

The FSS is a periodic structure of arranged metal patches or slits that functions as a spatial filter; i.e, it can realize strong transmission or reflection in a specific operating frequency band. Following decades of development, many basic units are in use; this section takes the basic square ring unit [31] as an example and uses the commercial solver CST to sweep the parameter (square ring size and substrate pitch) to obtain the S-band transmission and Ku-band reflection structure.

**Figure 8:**
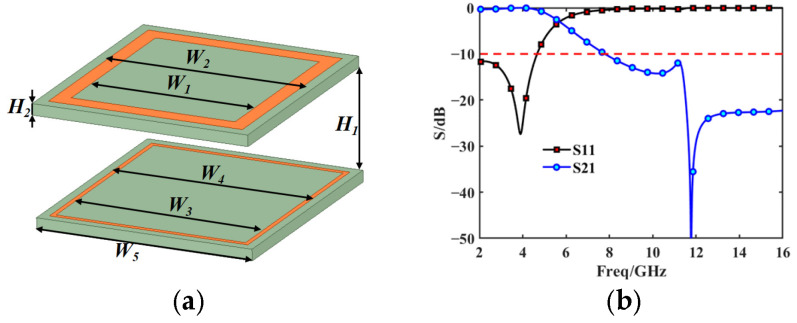
FSS structure and performance. (**a**) FSS model (**b**) Scattering parameters.

The proposed FSS consists of two layers of an equal-thickness substrate (Rogers 6002), which has a relative permittivity of 2.94 and a dielectric loss tangent of 0.0012. Two differently sized square ring metals were printed on both layers of the dielectric substrate, as shown in Figure 8a. The scattering parameters in Figure 8b show that this FSS achieves S-band transmission and Ku-band reflection and can achieve a low-frequency insertion loss of up to 0.5 dB in the S-band. At the same time, it is able to achieve at least 20 dB of high-frequency insertion loss (over 99% reflection) at 14–16 GHz. It is guaranteed that the electromagnetic wave can propagate in the mode shown in Figure 2. The specifically designed values are listed in Table 1. These value parameters were obtained by sweeping the parameters.

**Figure 9:**
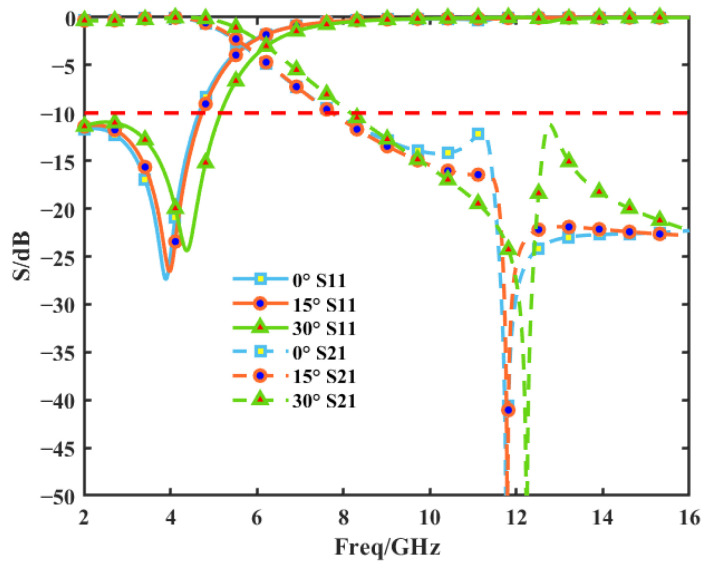
Scattering parameters of the proposed FSS structure for various incident angles.

Because the subreflector must be machined into a curved surface, the scattering performance of the FSS at various incident angles was examined. The results show that the FSS exhibits stable resonance behavior up to a 30° angle (as shown in Figure 9 for S-band transmission and Ku-band reflection). If the operating band needs to be adjusted, the value of H_1_ can be adjusted for the FSS structure, or it can be changed for other structures; e.g., it can be transmitted in the C-band and reflected in the Ku-band [32]. In addition, it has been shown in [23] that the FSS machined into a curved surface does not change the original radiation pattern of the antenna; therefore, the effect of the FSS having a curved surface on its performance is not considered in the subsequent sections of this paper. Based on the transmission and reflection properties of the FSS, it is ideally assumed that the antenna radiation can be transmitted or reflected by the FSS.

B. Shaped Design

In this design, for simplicity, both feeds are considered to be linearly polarized Gaussian feeds [33], with an irradiance level of −14 dB and irradiation angles of 64° and 44°, respectively. The main reflector aperture is 6.5 m, the ratio of the subreflector diameter to the main reflector diameter is 0.1, and the focal diameter ratio is 0.4. The shaped reflector antenna in this study is intentionally designed for narrowband single-frequency operation at 3.5 GHz and 14.5 GHz. According to Equation (8), the *D_λ_* of Beam I and Beam II are determined to be 38 dBi and 52 dBi, respectively. Here, in order to ensure that the antenna can meet the directivity requirements in both coverage areas, a smaller value is used for optimization.

The shaped design parameters *c_nm_* and *d_nm_* are set according to [26], as shown in Table 2, with a total of 27 optimization parameters. The optimization is then performed using PSO according to the process shown in Figure 5.

**Figure 10:**
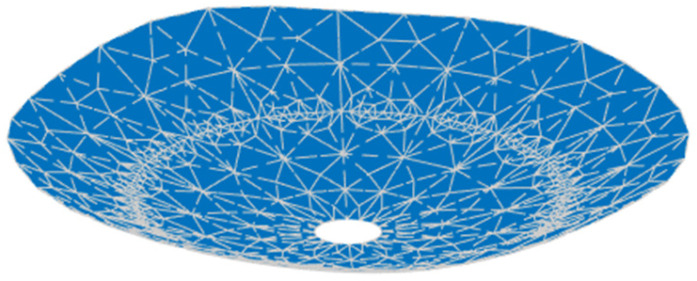
Shaped main reflector surface profile.

**Figure 11:**
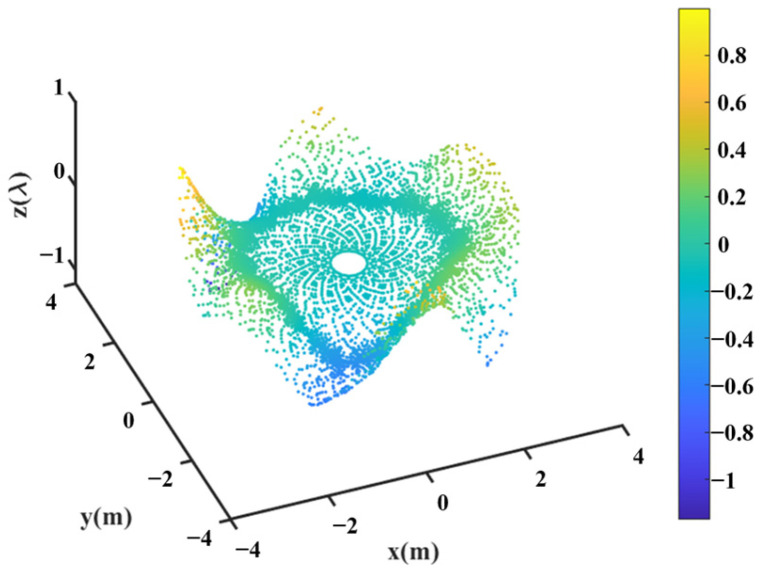
Deformation of the shaped main reflector.

Firstly, the main reflector is shaped at 3.5 GHz, and the design values of the parameters describing the shape of the main reflector surface were optimized, as shown in Table 3, and the shape of the main surface is shown in Figure 10. It can be observed that the shaped main reflector surface is still very smooth and can be further machined. Figure 11 shows the deformation of the shaped main reflector, from which it can be seen that the maximum deformation of the main reflector surface is less than 1λ. Please note that λ here is relative to 3.5 GHz.

**Figure 12:**
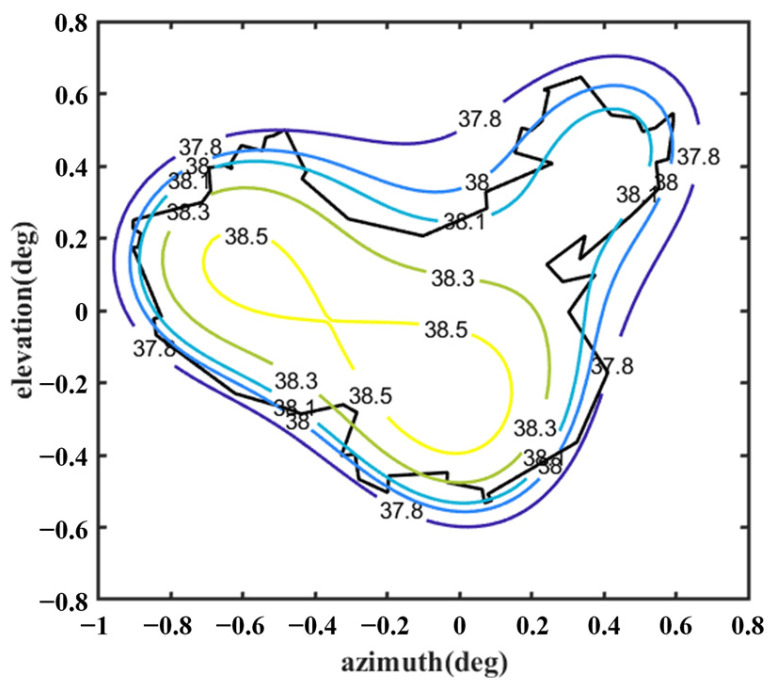
Far-field contour patterns of the optimized shaped main reflector antenna operating at 3.5 GHz illuminated by Feed 1 (note: optimized by Equation (9)).

**Figure 13:**
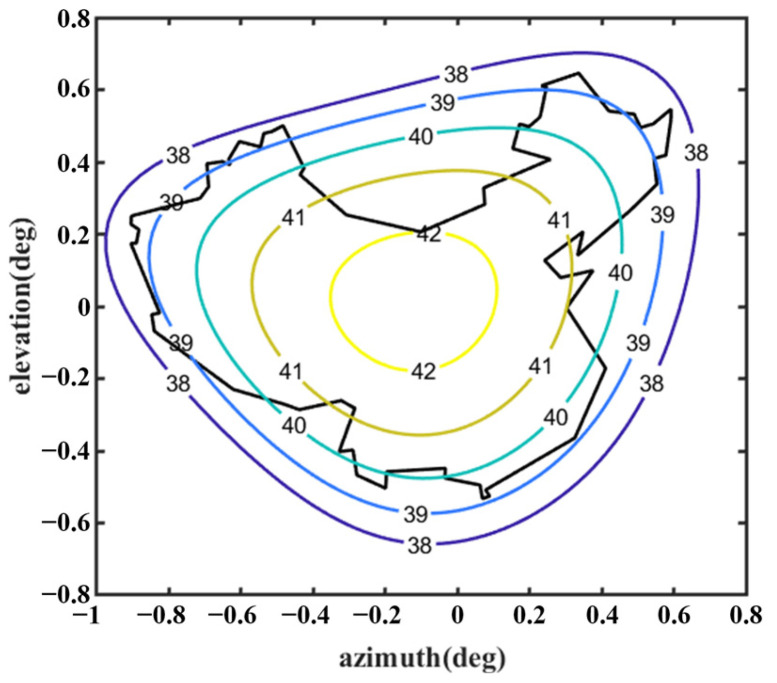
Far-field contour patterns of the optimized shaped main reflector antenna operating at 3.5 GHz illuminated by Feed 1 (note: optimized using Equation (10)).

**Figure 14:**
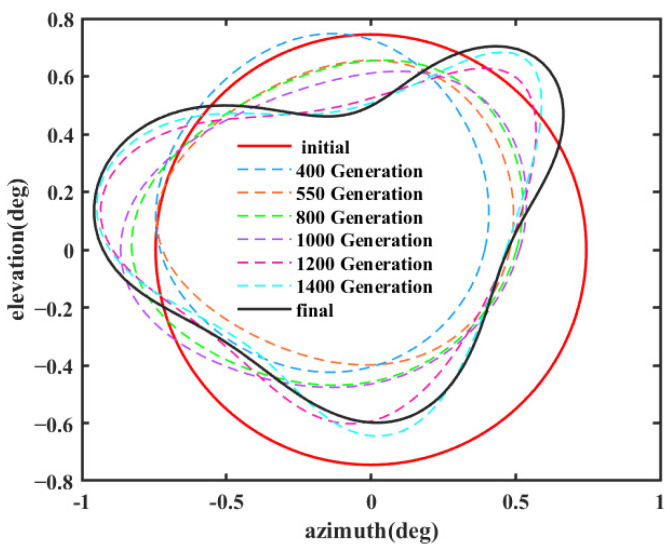
Contour beam iteration.

Figure 12 shows the optimized far-field contour patterns. It can be observed that the shaped contour of Beam I matches the shape of the desired regional contours. Please note that the far-field directivity of all observation sites meets the requirement of greater than 37.8 dBi in the coverage area of interest. This value is 0.2 dBi smaller than the design value of 38 dBi, but the field value changes more uniformly in the coverage area. The range of the field value variation is less than 1 dBi in the entire coverage area. Although the optimization result using Equation (10) without *D_u_* can satisfy the requirement of 38 dBi, the range of field value variation in the coverage area is much larger, and it does not match the boundaries of the coverage area very well (see Figure 13). Figure 14 shows the changes in the contour beam during the optimization process, clearly illustrating how the beam shape gradually approaches the designated area from its initial circular form.

**Figure 15:**
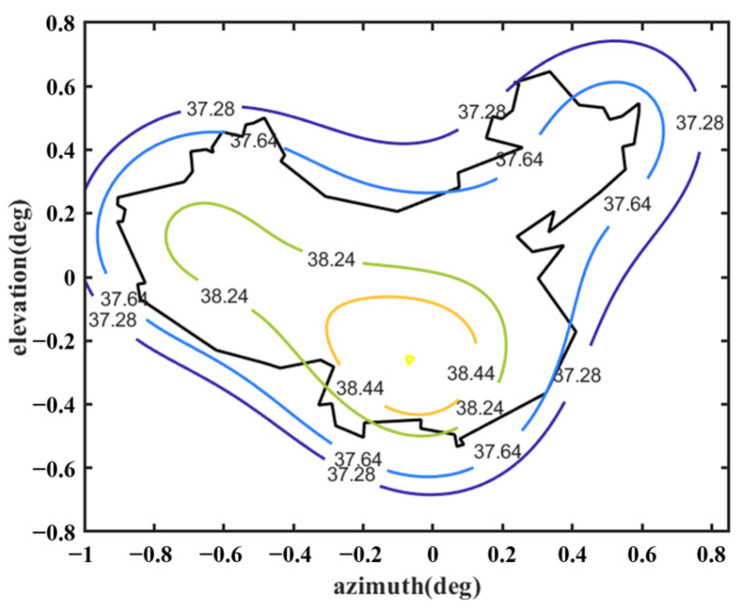
Far-field contour patterns of the optimized shaped main reflector antenna operating at 3.5 GHz illuminated by Feed 1 (calculated by GRASP).

Further, the shaped results were validated using GRASP, as shown in Figure 15. The results in Figure 12 and Figure 15 are very similar, and there is a difference of about 0.5 dBi between them at the boundary of the coverage area region, which is due to the fact that GRASP takes diffraction into account.

**Figure 16:**
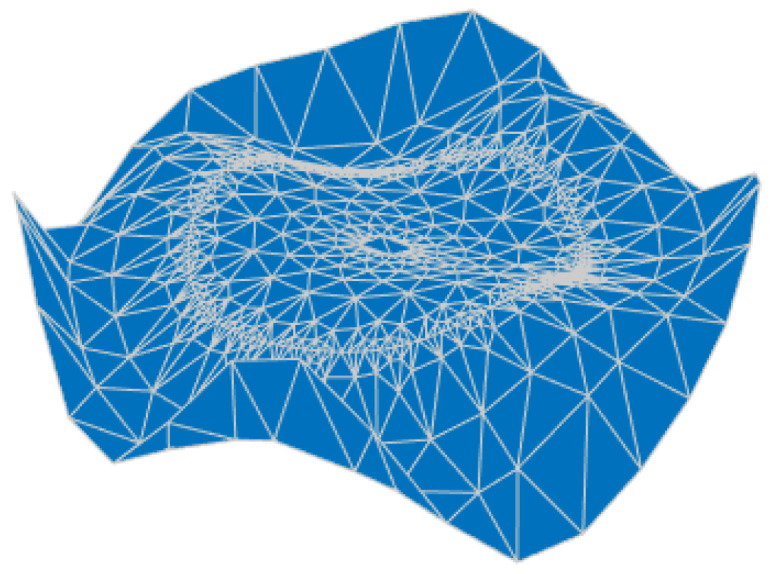
Shaped subreflector surface profile.

**Figure 17:**
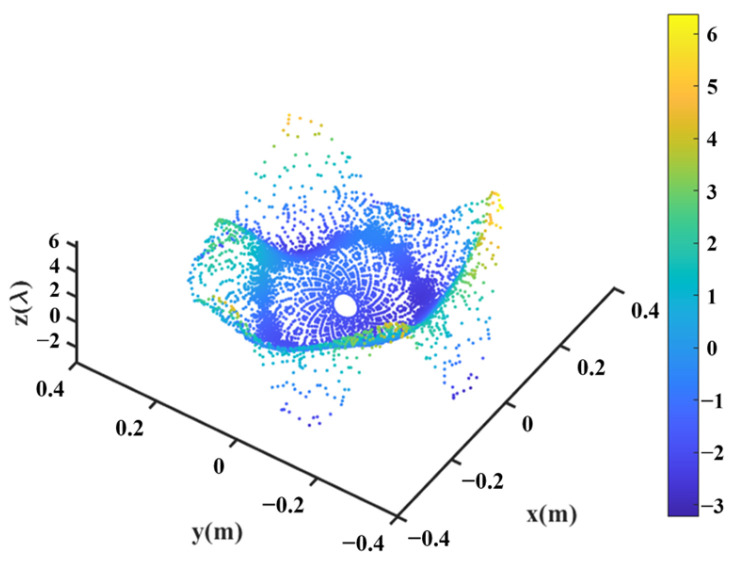
Deformation of shaped subreflector.

**Figure 18:**
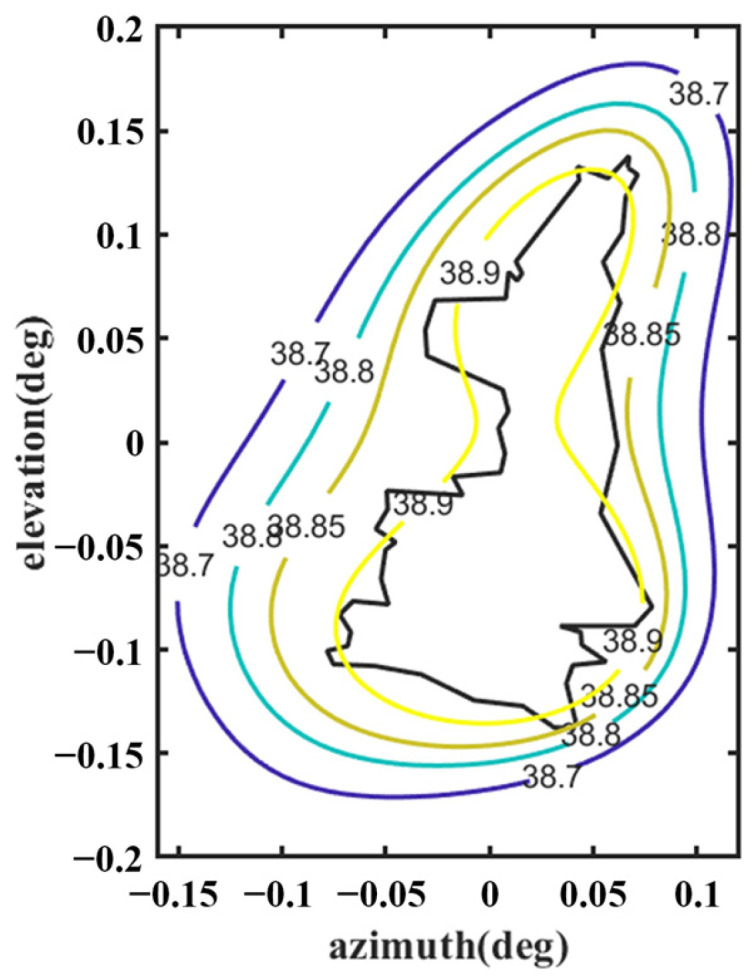
Far-field contour patterns of the optimized shaped main reflector antenna and subreflector operating at 14.5 GHz illuminated by Feed 2.

Keeping the surface of the shaped main reflector unchanged, the optimization continues following the process shown in Figure 5. The design values of the parameters describing the shape of the subreflector surface are optimized, as listed in Table 4. The shape of the subreflector is shown in Figure 16, and its diameter is 0.65 m. Similar to the main reflector surface, it is smooth, but its deformation is much greater. Figure 17 shows the deformation of the shaped subreflector, from which it can be seen that the maximum deformation is almost 6λ. Please note that λ here is relative to 14.5 GHz. Figure 18 shows the optimized far-field contour patterns. It can be observed that the shaped contour of Beam II matches the shape of the desired regional contours.

## 4. Conclusions

In this study, we propose a shared-aperture dual-band dual-beam reflector surface antenna design method assisted by frequency-selective subreflectors to provide contoured beam coverage in two different bands. On the one hand, the feasibility of the design is verified by extending the traditional shaped single-reflector design method to a dual-reflector surface by assigning shapes to the two reflectors to produce different beams covering both continental China and the Shaanxi area. On the other hand, by adding an extra constraint term, Du, to the optimization objective function, the designed beams produce more uniform field values in the coverage area.

This design concept provides a more flexible option for multibeam antennas. With the increase in satellite communication services, this shared-aperture-shaped-dual-reflector antenna has the potential to replace two large reflectors on spacecraft and can be widely used.

It should be noted that the current analysis assumes ideal FSS operating conditions. In real-world deployment scenarios, solar-induced thermal effects may cause structural deformation of the FSS, which affects its electromagnetic performance. As such, a comprehensive evaluation of multiple environmental factors, particularly the impact of solar radiation on structural performance, will be conducted in subsequent studies, such as incorporating thermally insensitive materials in the design.

## Figures and Tables

**Table 1:** Parameters of the FSS (mm).

W_1_	W_2_	W_3_	W_4_	W_5_	H_1_	H_2_
3.4	4.4	4.5	4.6	5	14.5	0.15

**Table 2:** Shaped the design parameters.

	n	0	1	2	3	4	5	6		
m										
0			*c*_10_	*c*_20_	*c*_30_	*c*_40_	*c*_50_	*c*_60_		
1		*c*_01_	*c*_12_	*c*_21_	*c*_31_	*c*_41_	*d*_22_	*d*_12_	2	
2		*c*_02_	*c*_13_	*c*_22_	*d*_41_	*d*_31_	*d*_21_	*d*_11_	1	
3		*c*_03_	*d*_60_	*d*_50_	*d*_40_	*d*_30_	*d*_20_	*d*_10_	0	
			6	5	4	3	2	1		m
									n	

**Table 3:** Optimized design parameter for main reflector surface (10^−3^).

	n	0	1	2	3	4	5	6		
m										
0			−2.5	−9.5	7.5	−5.7	−7.0	9.1		
1		−203	0.4	−0.9	1.9	3.0	−1.7	−0.1	2	
2		0.03	0.1	0.9	−4.3	4.5	3.7	−1.3	1	
3		−0.9	0.3	−0.9	1.6	−3.2	3.4	−1	0	
			6	5	4	3	2	1		m
									n	

**Table 4:** Optimized design parameter for subreflector surface (10^−3^).

	n	0	1	2	3	4	5	6		
m										
0			10	−10	10	−1.3	10	7.7		
1		−10.0	5.6	10	10	10	−10	0.5	2	
2		3.6	−9.4	−8.8	7.8	10	9.1	−1.0	1	
3		−3.3	10	−10	−1.6	−10	10	−10	0	
			6	5	4	3	2	1		m
									n	

## Data Availability

Data are contained within the article.
